# Supplementary material for: The interactome of intact mitochondria by cross-linking mass spectrometry provides evidence for coexisting respiratory supercomplexes
Source: Mol Cell Proteomics. 2017 Dec 8;17(2):216–32. doi: 10.1074/mcp.RA117.000470 (PMC5795388; doi:10.1074/mcp.RA117.000470)
Supplement: Supplemental Data [file supp_RA117.000470_133922_0_supp_23978_fzffwf.zip › spectra_annotation/mito_spectra_annotation/175-1-6-1-19-1.pdf]

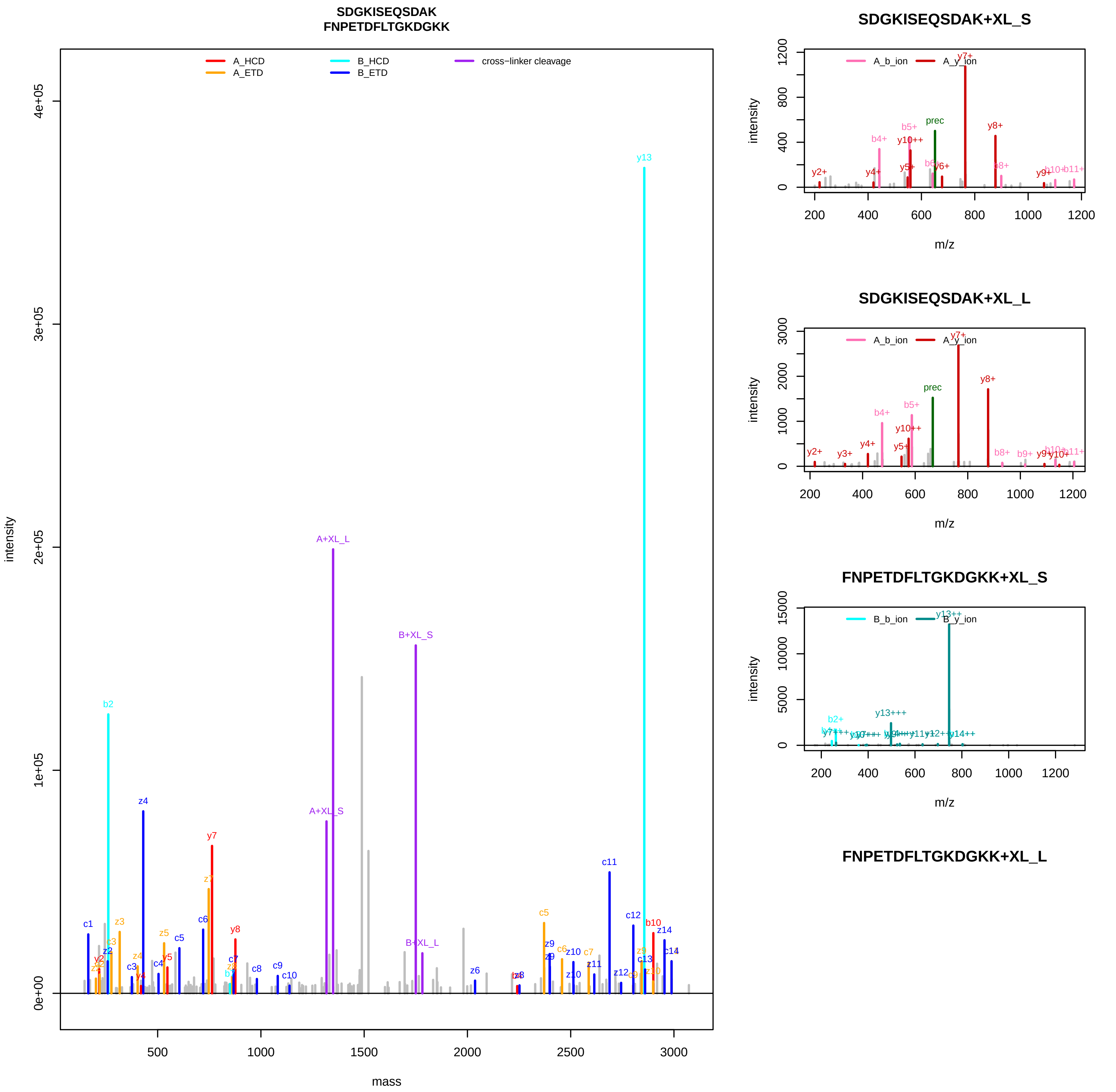
SDGKISEQSDAK
FNPETDFLTGKDGKK
A_HCD
A_ETD
B_HCD
B_ETD
cross−linker cleavage
intensity
mass
0e+00
1e+05
2e+05
3e+05
4e+05
500
1000
1500
2000
2500
3000
y13
A+XL_L
B+XL_S
A+XL_S
B+XL_L
b2
z4
y7
z7
c11
c12
b10
z14
c14
c1
c3
z3
z5
c5
c6
y8
c5
z9
z10
c7
z11
z12
c13
z6
c9
c10
c8
SDGKISEQSDAK+XL_S
A_b_ion
A_y_ion
y7+
y8+
prec
b5+
y10++
b4+
y4+
y5+
y6+
y2+
b8+
y9+
b10+
b11+
m/z
SDGKISEQSDAK+XL_L
y3+
b9+
FNPETDFLTGKDGKK+XL_S
B_b_ion
B_y_ion
y13++
y13+++
b2+
y14++
FNPETDFLTGKDGKK+XL_L
